# Multivariable Analysis of 169 Cases of Advanced Cutaneous Melanoma to Evaluate Antibiotic Exposure as Predictor of Survival to Anti-PD-1 Based Immunotherapies

**DOI:** 10.3390/antibiotics9110740

**Published:** 2020-10-27

**Authors:** Umang Swami, Adithya Chennamadhavuni, Nicholas Borcherding, Aaron D. Bossler, Sarah L. Mott, Rohan Garje, Yousef Zakharia, Mohammed Milhem

**Affiliations:** 1 Department of Internal Medicine, Division of Oncology, Huntsman Cancer Institute, University of Utah, Salt Lake City, UT 84112, USA; 2 Holden Comprehensive Cancer Center, University of Iowa, Iowa City, IA 52242, USA; adithya-chennamadhavuni@uiowa.edu (A.C.); nicholas-borcherding@uiowa.edu (N.B.); sarah-mott@uiowa.edu (S.L.M.); rohan-garje@uiowa.edu (R.G.); yousef-zakharia@uiowa.edu (Y.Z.); mohammed-milhem@uiowa.edu (M.M.); 3 Department of Pathology, University of Iowa Hospitals and Clinics, 200 Hawkins Dr., Iowa City, IA 52242, USA; aaron-bossler@uiowa.edu

**Keywords:** melanoma, microbiome, antibiotics, microbiota, PD-1

## Abstract

Recently antibiotic exposure has been associated with worse outcomes in patients undergoing treatment with antibodies directed against programmed cell death protein-1 (PD-1). We reviewed data of 1264 patients enrolled at Melanoma Skin and Ocular Tissue Repositories at University of Iowa Hospitals and Clinic. Reviewed data included patient demographics, prior medical history, baseline hematologic and disease parameters and outcomes including progression-free survival (PFS) and overall survival (OS). Cox regression models were used to determine predictive markers. Overall, 169 patients with advanced cutaneous melanoma received anti-PD-1 based therapies. Median follow up was 18.46 (range 0.89 to 62.52) months. On multivariable analysis brain metastasis, higher absolute neutrophil count (ANC) and lower absolute lymphocyte count were associated with poorer PFS while brain and liver metastasis and lower albumin were associated with poorer OS. Prior antibiotics, radiation as well as age, gender, basal metabolic index (BMI), smoking status, *BRAF* mutation, line of therapy (first or latter), prior treatments (ipilimumab or BRAF inhibitors), hemoglobin, neutrophil-to-lymphocyte ratio, white blood cell, platelet and eosinophil counts were not associated with PFS or OS in multivariable analysis. Contrary to some prior studies BMI, radiation, and antibiotics were not associated with PFS or OS.

## 1. Introduction

Management of metastatic melanoma has undergone a tectonic shift with the advent of immunotherapies and targeted therapies. Since 2011, 10 agents have been approved for the treatment of metastatic melanoma including two programmed cell death protein-1 (PD-1) antibodies, pembrolizumab and nivolumab, which are approved for treatment of metastatic melanoma as front-line therapy [1]. These agents have significantly improved the historical median overall survival (OS) of patients with metastatic melanoma from 6.2 months [2] to more than 3 years [3]. However, progression-free survival (PFS) using anti-PD-1 therapies has remained poor, averaging 4 to 7 months with an overall response rate ranging from 27% to 44% [1]. Though anti-PD-1 therapies have been approved for all patients with metastatic melanoma regardless of PD-L1 expression, it is not known which patients will ultimately derive benefit from them. Therefore, a great deal of effort is being made to identify predictive biomarkers including evaluation of PD-L1 expression, tumor infiltrating lymphocytes, tumor mutational burden, microsatellite instability, mismatch-repair deficiency, neoantigen load, gene expression signatures, T-cell receptor diversity, and clonality, circulating immune-cell subsets, serum protein signatures, soluble PD-L1, gut microbiome, human leukocyte antigen genotype, and germline single-nucleotide polymorphism [4].

Identification of clinical predictors for response to anti-PD-1 therapies could benefit standard of care investigations (clinical, radiological, laboratory, and pathological) and may be more cost-effective, easy to interpret and use less resources and time. Over the last few years, a great emphasis has been placed on understanding the gut microbiome and its effect on modulation of response to immunotherapies. Results from preclinical and some clinical studies have raised the possibility that dysbiosis due to antibiotic use can decrease efficacy to immune checkpoint inhibitors [5,6]. However, other studies did not show correlation between outcomes with immune checkpoint inhibitors and prior antibiotics [7,8,9,10]. Therefore, due to conflicting evidence we aimed to identify the effect of antibiotics in our institutional cohort of patients with metastatic melanoma treated with anti-PD-1 therapies.

## 2. Materials and Methods

Data of 1264 patients enrolled at Melanoma Skin and Ocular Tissue Repositories at Holden Comprehensive Cancer Center at University of Iowa Hospitals and Clinics from 1 August 2012 to 31 July 2017 was reviewed to identify patients with unresectable, advanced, or metastatic cutaneous melanomas who received anti-PD-1 therapies. Reviewed data included demographics (gender, race, ethnicity), *BRAF* mutational status, smoking status, prior tanning bed usage, prior history of different cancer, performance status, body mass index (BMI), prior treatment regimens, baseline hematological parameters (complete blood count with differential, albumin, lactate dehydrogenase), radiation therapy 3 months prior to starting treatment, antibiotic exposure 2 months prior to starting anti PD-1 therapy and melanoma metastases to brain and liver. Identified patients were followed until 21 November 2018. Progression (clinical or radiological) and responses were determined by iRECIST and clinic notes [11]. Outcomes with anti-PD-1 therapies including PFS and OS were collected. The study was approved by the Institutional Review Board of University of Iowa Hospitals and Clinics.

### Statistical Analysis

Cox regression models were used to assess the effects of clinical and pathologic variables on PFS and OS. Time was calculated from initiation of PD-1 treatment to progression or death due to any cause for PFS, and to death due to any cause for OS. Using a stepwise selection procedure, variables with *p*-values < 0.10 at the univariable level were considered for inclusion in their respective multivariable model. Estimated effects of predictors are reported as hazard ratios (HR) along with 95% confidence intervals (CI). All statistical testing was two-sided and assessed for significance at the 5% level using SAS v9.4 (SAS Institute, Cary, NC, USA).

## 3. Results

Of patients with advanced, unresectable, or metastatic cutaneous (including one pineal gland) melanoma there were 169 total who received pembrolizumab- or nivolumab-based therapies, 100 of whom received anti-PD-1 therapies as first line therapy and 111 who received it as a single agent. The baseline demographics and patient characteristics are presented in Table 1 and Table 2. Median age was 63 (range 24 to 98) years and median follow up was 18.46 (range 0.89 to 62.52) months. Thirty-nine patients had brain metastasis and 29 had liver metastasis prior to starting anti-PD-1 therapy. With treatment 46 (27.5%) patients had complete response, 30 (18.0%) had partial response, 34 (20.4%) had stable disease, and 57 (34.1%) had progressive disease. Response assessments were missing for two patients. 

**Figure 1:**
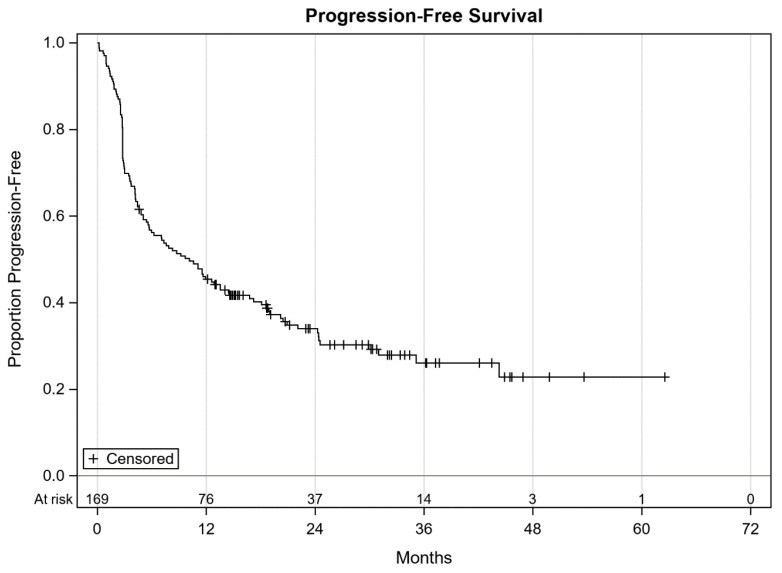
Progression-free survival of all patients with cutaneous melanoma.

**Figure 2:**
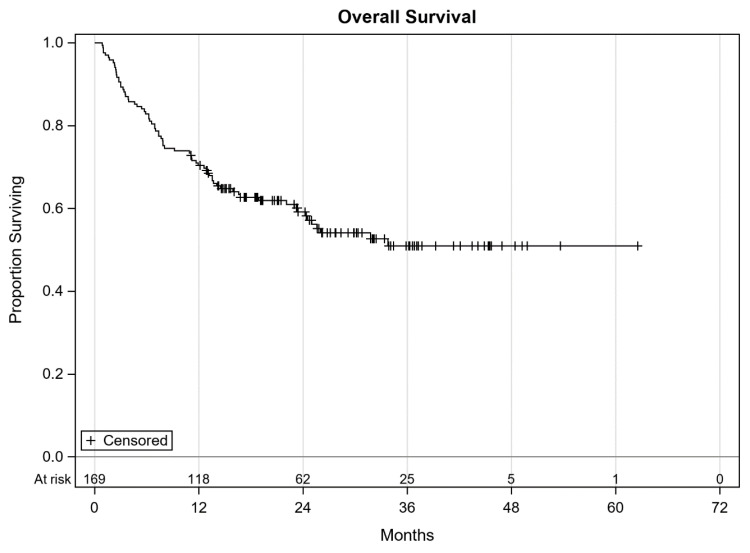
Overall survival of all patients with cutaneous melanoma.

On follow-up, 108 (63.9%) patients were found to have progressed while the rest continued without progression. At the time of data cut-off, 96 (56.8%) patients were alive and the remaining 73 (43.2%) were dead. Overall, 150 (88.8%) patients had discontinued the study treatment. Reasons for treatment discontinuation included progression (85 patients), immune related adverse events (28 patients), derivation of maximum benefit per investigator and/or patient (23 patients), and other reasons such as decline in performance status, insurance denials, patient choice, development of another malignancy, death due to other causes etc. (14 patients). The median PFS was 10.1 months (95% CI 5.7–14.5, Figure 1). Median OS was not reached likely due to a short follow-up (Figure 2). 

At the time of univariable and multivariable analysis history of tanning bed use and baseline LDH were removed due to high rate of missing data. Univariable analysis showed poorer PFS with the features of brain metastasis, elevated white blood cell (WBC) counts, absolute neutrophil (ANC), neutrophil to lymphocyte ratio (NLR), and low albumin (Table 3), and showed poorer OS associated with the features of brain metastasis, liver metastasis, radiation treatment within prior 3 months, and antibiotics within prior 2 months of starting anti-PD-1 based therapies along with elevated WBC, ANC, NLR and a lower hemoglobin, and albumin (Table 4). 

**Figure 3:**
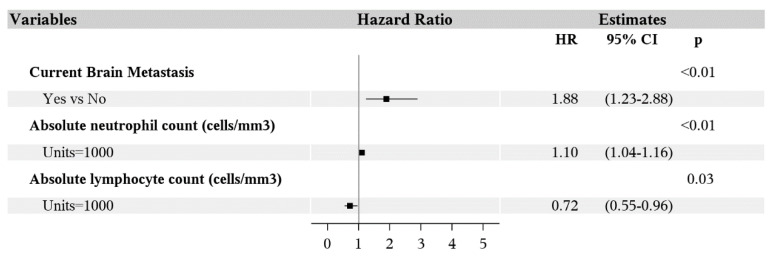
Forest plot of multivariable analysis for predictors of progression-free survival.

**Figure 4:**
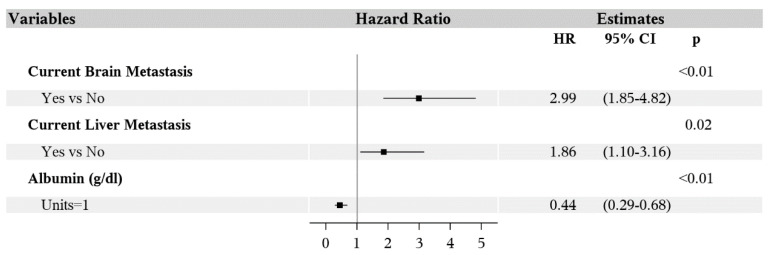
Forest plot of multivariable analysis for predictors of overall survival.

Multivariable analysis showed poorer PFS was associated with the features of brain metastasis (HR 1.88; 95% CI 1.23–2.88; *p* < 0.01), higher ANC (HR 1.10; 95% CI 1.04–1.16; *p* < 0.01) and lower absolute lymphocyte count (ALC, HR 0.72; 95% CI 0.55–0.96; *p* = 0.03) (Figure 3). Overall survival was worse for patients with history of brain metastasis (HR 2.99; 95% CI 1.85–4.82; *p* < 0.01), liver metastasis (HR 1.86; 95% CI 1.10–3.16; *p* = 0.02) and lower albumin (HR 0.44; 95% CI 0.29–0.68; *p* < 0.01) (Figure 4). Age, gender, BMI, smoking status, prior cancer, *BRAF* mutation, line of therapy (first or latter), prior treatment with ipilimumab or BRAF inhibitors, radiation, antibiotics, WBC, hemoglobin, platelet count, and eosinophil count were not associated with PFS or OS in the multivariable analysis.

## 4. Discussion

Our study presents a detailed analysis of various baseline variables which are part of standard of care to determine clinical predictors to anti-PD-1 therapies. As expected, metastasis to the brain was associated with worse PFS and OS. Similarly, liver metastasis and low albumin are predictors of worse OS. Prior meta-analysis and pooled analysis have shown that brain and liver metastasis are prognostic of poor survival [2,12] and low albumin may be an indirect marker of overall poor performance status and liver metastasis. 

Systemic inflammation has been implicated as a promoter of tumor development, progression and metastasis [13]. On univariable analysis elevated WBC and ANC were associated with poor PFS and OS. On multivariable analysis the association of elevated ANC with poor PFS was retained and became significant for low ALC also. However, the association was not seen between ANC and ALC with OS on multivariable analysis which might need a bigger data set to be evident. In a meta-analysis of 4,593 patients with melanoma, elevated NLR has been associated with a poor PFS (HR = 1.86; 95% CI = 1.24–2.80; *p* = 0.003) and OS (HR: 1.56, 95% CI: 1.28–1.90, *p* < 0.001) [13]. On univariable analysis, similar results were found for NLR > 4 being associated with OS and PFS; however, NLR was not retained in the multivariable models. 

Gut microbiome has shown to modulate response to anti-PD-1 therapies in preclinical models as well as melanoma patients [14] and therefore it is logical to assume that antibiotics too can alter the response by modulating the gut microbiome. Prior studies with checkpoint inhibitors have given varying results. For example, antibiotics have been associated with reduced benefit in melanoma [15,16], non-small cell lung cancer (NSCLC) [15,17,18], and kidney cancer [17]. However, other studies such as in NSCLC [7,8,9] and urothelial carcinoma [10] did not show an association of prior antibiotics with poor outcomes. In our study antibiotics did not affect the PFS and OS on multivariable analysis in patients with melanoma. In an earlier review we have discussed how the same datasets can give contrasting results based on different cut-offs for duration of antibiotics [5]. Due to our incomplete understanding of gut microbiome, the differential effect of various classes of antibiotics on gut microbiota and its timing in relation to anti-PD-1 therapy administration we can have different results in studies even with the same data-sets depending upon how they are analyzed [5,6].

Radiation has been shown to release damage-associated molecular patterns, toll-like receptors, increase expression of major histocompatibility complex (MHC) class I antigen and tumor-associated antigens, all of which can potentially contribute to synergy with immunotherapy as well as translate in abscopal effect [19]. However, our analysis did not reveal that radiation was a predictor of benefit. This might simply be because these patients received radiation due to brain metastasis or symptomatic disease and not primarily to sensitize and enhance anti-PD-1 activity. These results are in agreement with a recently conducted randomized trial of nivolumab with or without stereotactic body radiotherapy in patients with metastatic head and neck squamous cell carcinoma which did not show any difference in response or survival outcomes with addition of stereotactic body radiotherapy [20].

Our study also did not show an association of benefit with BMI which was seen in a recent pooled multi-cohort analysis of patients with metastatic melanoma. This might be reflective of our small sample size or different statistical design [21]. Our study does have similar limitations as most retrospective studies including missing data, chances of wrong coding, and confounding. It is also possible that our results are reflective of prognostic nature of variables in patients with metastatic melanoma. 

In conclusion, we found that a higher ANC, lower ALC, and brain metastasis were associated with poorer PFS while low albumin, brain metastasis, and liver metastasis were associated with poorer OS. We suggest treating these patients with a more aggressive approach including clinical trials with novel drugs and combinations to improve their outcomes. More preclinical and clinical efforts need to be made to understand the tumor biology and biomarkers which can predict benefit and resistance to various therapies. 

## Figures and Tables

**Table 1:** Baseline demographics and variables of patients with cutaneous melanoma.

Variable	Level	*N* = 169	%
Gender	Female	59	34.9
	Male	110	65.1
Race	White	168	100.0
	Missing	1	-
Ethnicity	Non-Hispanic	169	100.0
Smoking Status	Current	32	18.9
	Former	67	39.6
	Never	70	41.4
History of another cancer	No	159	94.1
	Yes	10	5.9
Performance Status	0	74	64.3
	1	37	32.2
	2	4	3.5
	Missing	54	-
Brain Metastasis	No	130	76.9
	Yes	39	23.1
Liver Metastasis	No	140	82.8
	Yes	29	17.2
*BRAF* Mutation	No	76	50.0
	Yes	76	50.0
	Missing	17	-
Prior Ipilimumab	No	114	67.9
	Yes	54	32.1
	Missing	1	-
Prior BRAF inhibitors	No	149	88.7
	Yes	19	11.3
	Missing	1	-
Radiation (within previous 3 months)	No	143	84.6
	Yes	26	15.4
Antibiotics (within previous 2 months)	No	136	81.9
	Yes	30	18.1
	Missing	3	-
Line of Therapy	First	100	59.2
	Second	49	29.0
	Third	15	8.9
	Fourth	5	3.0
Concurrent Radiation	No	147	87.0
	Yes	22	13.0
Regimen	Combination	58	34.3
	Single	111	65.7
Neutrophil to lymphocyte ratio	≤4	113	68.5
	>4	52	31.5
	Missing	4	-

**Table 2:** Baseline variables and their distribution.

Variable	*N*	Missing	Minimum	Maximum	Median	Mean	Standard Deviation
Age (years)	169	0	24.00	98.00	63.00	62.12	15.74
Body mass index	166	3	17.35	60.52	28.32	29.40	6.39
White blood cells (1000/mm^3^)	167	2	2.30	52.00	7.10	8.30	4.64
Hemoglobin (g/dL)	167	2	7.20	18.10	13.60	13.30	2.00
Platelets (1000/mm^3^)	166	3	93.00	711.00	236.00	255.77	93.93
Absolute neutrophil count (cells/mm^3^)	165	4	4.94	41,590.00	4650.00	5601.81	3928.37
Absolute lymphocyte count (cells/mm^3^)	165	4	2.02	4680.00	1524.00	1625.87	748.77
Eosinophils (cells/mm^3^)	164	5	0.00	3120.00	180.00	236.43	297.53
Albumin	166	3	2.30	5.00	4.10	4.01	0.49
Duration of Anti-PD-1 Therapy (months)	169	0	0.07	62.52	5.59	9.68	9.82
Length of follow-up (months)	169	0	0.89	62.52	18.46	20.08	13.55

**Table 3:** Univariable analysis for predictors of progression-free survival.

Covariate	Level	*N*	Progression-Free Survival			
			Hazard Ratio	95% CI		*p*-Value
Gender	Female	59	1.09	0.75	1.60	0.65
	Male	110	Ref	-	-	
Smoking Status	Current	32	1.03	0.60	1.77	0.80
	Former	67	1.14	0.77	1.70	
	Never	70	Ref	-	-	
History of another cancer	Yes	10	0.90	0.42	1.93	0.78
	No	159	Ref	-	-	
Brain Metastasis	Yes	39	1.84	1.22	2.76	<0.01
	No	130	Ref	-	-	
Liver Metastasis	Yes	29	1.47	0.91	2.36	0.11
	No	140	Ref	-	-	
*BRAF* Mutation	Yes	76	0.84	0.58	1.22	0.36
	No	76	Ref	-	-	
Prior Systemic Therapy	Yes	69	0.94	0.64	1.36	0.73
	No	100	Ref	-	-	
Prior Ipilimumab	Yes	54	0.95	0.64	1.41	0.81
	No	114	Ref	-	-	
Prior BRAF inhibitors	Yes	19	0.96	0.54	1.72	0.90
	No	149	Ref	-	-	
Radiation (Within Previous 3 Months)	Yes	26	1.60	1.00	2.58	0.05
	No	143	Ref	-	-	
Antibiotics (Within Previous 2 months)	Yes	30	1.28	0.80	2.04	0.30
	No	136	Ref	-	-	
Neutrophil to lymphocyte ratio	>4	52	1.70	1.16	2.50	<0.01
	≤4	113	Ref	-	-	
Age (years)	Units = 10	169	1.05	0.93	1.17	0.46
Body mass index	Units = 5	166	1.04	0.89	1.20	0.64
White blood cells (1000/mm^3^)	Units = 1	167	1.08	1.03	1.13	<0.01
Hemoglobin (g/dL)	Units = 1	167	0.98	0.89	1.07	0.63
Platelets (1000/mm^3^)	Units = 100	166	1.07	0.88	1.29	0.52
Absolute neutrophil count (cells/mm^3^)	Units = 1000	165	1.11	1.05	1.17	<0.01
Absolute lymphocyte count (cells/mm^3^)	Units = 1000	165	0.78	0.59	1.04	0.08
Eosinophils (cells/mm^3^)	Units = 100	164	1.00	0.91	1.09	0.92
Albumin (g/dL)	Units = 1	166	0.63	0.44	0.89	<0.01

**Table 4:** Univariable analysis for predictors of overall survival.

Covariate	Level	*N*	Overall Survival			
			Hazard Ratio	95% CI		*p*-Value
Gender	Female	59	0.98	0.60	1.59	0.93
	Male	110	Ref	-	-	
Smoking Status	Current	32	1.25	0.66	2.36	0.79
	Former	67	1.06	0.63	1.76	
	Never	70	Ref	-	-	
History of another cancer	Yes	10	0.57	0.18	1.82	0.35
	No	159	Ref	-	-	
Brain metastasis	Yes	39	3.41	2.13	5.46	<0.01
	No	130	Ref	-	-	
Liver metastasis	Yes	29	2.06	1.22	3.48	<0.01
	No	140	Ref	-	-	
*BRAF* Mutation	Yes	76	0.74	0.46	1.20	0.22
	No	76	Ref	-	-	
Prior Systemic Therapy	Yes	69	1.14	0.72	1.82	0.57
	No	100	Ref	-	-	
Prior Ipilimumab	Yes	54	1.16	0.71	1.88	0.55
	No	114	Ref	-	-	
Prior BRAF inhibitors	Yes	19	1.36	0.70	2.66	0.37
	No	149	Ref	-	-	
Radiation (Within Previous 3 Months)	Yes	26	2.35	1.36	4.06	<0.01
	No	143	Ref	-	-	
Antibiotics (Within Previous 2 months)	Yes	30	1.73	1.00	2.99	0.05
	No	136	Ref	-	-	
Neutrophil to lymphocyte ratio	>4	52	2.28	1.42	3.63	<0.01
	≤4	113	Ref	-	-	
Age (years)	Units = 10	169	1.11	0.95	1.29	0.19
Body mass index	Units = 5	166	0.98	0.81	1.19	0.84
White blood cells (1000/mm^3^)	Units = 1	167	1.07	1.03	1.12	<0.01
Hemoglobin (g/dL)	Units = 1	167	0.89	0.79	0.99	0.04
Platelets (1000/mm^3^)	Units = 100	166	1.12	0.87	1.43	0.38
Absolute neutrophil count (cells/mm^3^)	Units = 1000	165	1.10	1.05	1.15	<0.01
Absolute lymphocyte count (cells/mm^3^)	Units = 1000	165	0.78	0.55	1.10	0.16
Eosinophils (cells/mm^3^)	Units = 100	164	0.99	0.88	1.11	0.80
Albumin (g/dL)	Units = 1	166	0.42	0.28	0.62	<0.01
